# Feedback and Trigger of Household Decision-Making to Ecological Protection Policies in Sanjiangyuan National Park

**DOI:** 10.3389/fpls.2021.827618

**Published:** 2022-01-17

**Authors:** Xukun Su, Yu Shen, Shikui Dong, Yuqing Liu, Hao Cheng, Lingfan Wan, Guohua Liu

**Affiliations:** ^1^State Key Laboratory of Urban and Regional Ecology, Research Center for Eco-Environmental Sciences, Chinese Academy of Sciences, Beijing, China; ^2^College of Resources and Environment, University of Chinese Academy of Sciences, Beijing, China; ^3^College of Grassland Science, Beijing Forestry University, Beijing, China

**Keywords:** alpine grassland, G-Range model, DECUMA model, household decision-making, local herdsmen, Sanjiangyuan National Park

## Abstract

Ignoring the responses of local households to ecological protection policies can not only seriously limit sustainable development of the alpine grassland ecosystem, but also not improve livelihood on the Qinghai-Tibetan Plateau (QTP). It is of vital importance to clearly understand coupling feedback and trigger between household decision-making of local herdsmen with the implementation of ecological protection policies. We selected Sanjiangyuan National Park (SNP) as the study area which was in the hinterland of the QTP and the first national park in China. We used the global rangeland (G-Range) model to simulate alpine grassland changes and DEcisions under Conditions of Uncertainty by Modeled Agents (DECUMA) model to identify household decision-making of local herdsmen. Results showed that: (1) distribution of livestock density was basically consistent with the distribution of habitat suitability of local households in the SNP; (2) more than half of the uneducated households (52 and 70%) opposed the eco-compensation and eco-migration policies; (3) most of the households (53.7%) never traded livestock for maintaining their livelihood; and (4) When local households owed 65,000 yuan (≈10,000 dollars) in debts, as the critical value (trigger), they traded livestock to support their livelihood. We suggest that feedback and trigger of household decision-making should be fully considered by managers of national park and policymakers of local governments in planning ecological protection policies to maintain sustainable development of alpine grassland, which is of practical significance to long-term conservation and sustainable utilization of natural resources in the SNP.

## Highlights

- Spatial distributions of livestock density and habitat suitability of local households were basically consistent in the Sanjiangyuan National Park.
- Most of the local households (nearly 70%) opposed eco-migration policy.
- When local households owed 65,000 yuan (≈10,000 dollars) in debts as the trigger, they traded livestock to support their livelihood.

## Introduction

The Intergovernmental Platform on Biodiversity and Ecosystem Services (IPBES) aims to establish links between scientific research and policymaking to enhance the conservation and utilization of biodiversity and to ensure long-term human wellbeing and the sustainable development of societies (Wan et al., 2021). Recently, a series of assessments from the IPBES show that sustainable goals may be likely to be achieved with an improved understanding of the feedback relationship between society and ecosystems, in global, while improved effectiveness of governance systems also matters (Mastrangelo et al., 2019; Ma et al., 2020). For obtaining more wellbeing from ecosystems, humans alter natural ecosystems, either directly or indirectly (Thiault et al., 2018). Due to the high intensity of human activities, the authenticity of natural ecosystems has deviated over the past few decades (Díaz et al., 2019; Zhao et al., 2020). Humans depend on and mainly drive both changes of ecosystems and their services. People obtain various benefits from ecosystems by harvesting food, earning income, gaining protection, or deriving social and cultural meaning, which underpin human wellbeing (Thiault et al., 2018). Dealing with social and ecological problems has puzzled many researchers for a long time (Liu et al., 2016). It is the overarching challenge that relationships between human wellbeing and ecosystem conservation are difficult to integrate into tools to effectively guide decision-making (Thiault et al., 2018). Maintaining sustainable development and improving human wellbeing have been highly considered by policymakers in China. The long-term social and ecological protection policies and plans have significantly contributed to maintain local ecological stability (Ma et al., 2020).

Natural reserves are the cornerstone of biodiversity conservation. Enhancement of human wellbeing and protection of natural ecosystems in natural reserves have been of international concerns on sustainable development (Fang, 2013; Mastrangelo et al., 2019; Zheng et al., 2020). Of all the effort, natural reserves have been the most prevalent approach shared by governments in over 150 countries (Ma et al., 2020). The report of the 19th National Congress of the Communist Party of China has explicitly proposed to “construction of protected areas system with national parks as the main body” which is a major measure of ecological civilization of China (Du et al., 2020; Gu et al., 2020; Zhao, 2021). Its targets are to protect the authenticity and integrity of the ecosystems and to achieve the harmonious development between humans and nature (Zhao et al., 2020; Zhao, 2021). Construction of national parks in China is still in the experimental stage, requiring further progress and following a top-down management model (Ma et al., 2020). As the primary type of protected area, national park has been playing an important role in biodiversity conservation (Ma et al., 2020). As the first national park in China, the establishment of Sanjiangyuan National Park (SNP) on the Qinghai-Tibetan Plateau (QTP) brings a sustainability focus and is vital for wildlife protection with respect to both phylogenetic resources and conservation efforts (Lin and Zhang, 2020; Xi et al., 2020; Su et al., 2021). Alpine grassland, which is the main ecosystem in the SNP, can provide multiple ecosystem services which contain provision services (e.g., food provision to wildlife and livestock), regulatory services (e.g., flood and disease control), cultural services (e.g., spiritual, recreational, and cultural benefits), and support services (e.g., ecosystem nutrient cycling) (Wang et al., 2016). The SNP is also the main habitat of endemic wildlife and livestock, and the local people depend on these special animals for survival and development (Shen and Tan, 2012; Han et al., 2018; Dai et al., 2019; Wang et al., 2020). A total of 22 ecological restoration strategies have been implemented for more than 20 years (Wang et al., 2016; Sheng et al., 2019). Aiming to reduce ecosystem degradation, alleviating rural poverty with central government supports, and promoting local sustainable development of economy, these plans bring enormous social and ecological benefits to the SNP (Ma et al., 2020). Local households obtain almost all their living necessities from grazing their livestock, such as yaks, Tibetan sheep, and horses in the SNP (Wang et al., 2016). Historically, they migrated in long distances from summer pastures to winter pastures to graze more livestock and to avoid loss from frequent natural disasters, especially snowstorms (Wang et al., 2016). It can be understood as an integrated socio-ecological system that local households, their livestock, and alpine grassland ecosystem develop interaction between feedbacks after thousands of years of adaptation (Wang et al., 2016; Yeh et al., 2017). The traditional lifestyle of local households is the result of a long period of adaptation to the harsh climatic and geographical characteristics of the QTP (Wang et al., 2016). Local households are the dominant and direct beneficiaries of the alpine grassland ecosystem, and their feedbacks and decision-making determine whether the alpine grassland ecosystem can be sustainable development in the future (Yang et al., 2020). Meanwhile, grassland ecosystem also affects the feedback and decision-making of local households, such as the quality of herbage and water supply (Daily and Matson, 2008; Zheng et al., 2020). Current research on ecological protection behaviors of residents commonly adopts the socioeconomic approach at the individual level, which might ignore the impacts of potential psychological factors on resident behaviors, such as willingness and perception of farmers to conservation (Ma et al., 2020). Eco-compensation and eco-migration are two main grassland protection policies on the QTP. Eco-compensation is an institutional arrangement of rules, which aims to protect and sustainably use alpine grassland, adjusts the interests of local households mainly by economic means, promotes compensation activities, and arouses the enthusiasm of ecological protection (Zhao, 2021). Eco-migration policy, also known as environmental migration, refers to people who move out of their original residence in protected areas, areas severely damaged by ecological environment, ecologically fragile areas, and areas with poor natural environment and no living conditions for human beings, and who settle and rebuild their homes in other places (Zhai et al., 2020; Zhao, 2021).

Ignoring the responses and decision-making of local households to protection policies will seriously affect and directly determine the development direction of the local ecosystems in the future. It eventually leads to that efficiency of protection policies is not significant to maintain biodiversity and cannot fundamentally alleviate alpine grassland degradation in the SNP (Guo et al., 2021). It also cannot improve the livelihood and wellbeing of local households and ultimately cannot realize the sustainable utilization of alpine grassland ecosystem in the SNP (Wu et al., 2020). Household decision-making of local herdsmen should be an important part of the management and utilization of ecological environment in the SNP (Wan et al., 2021). It should be fully considered in planning sustainable development management policies which are of practical significance to the long-term protection and sustainable utilization of natural resources in the SNP. Therefore, there is an urgent need to use new methods to research on response of household decision-making to protection policies, coupling natural ecological and social-economic systems.

Aiming to clearly understand coupling relationships and feedback mechanisms of household decision-making to ecological protection policies, through using global rangeland (G-Range) model to simulate alpine grassland changes and DEcisions under Conditions of Uncertainty by Modeled Agents (DECUMA) model to identify household decision-making of local herdsmen, we documented: (1) habitat suitability changes of local households and spatial distributions of livestock density from 2000 to 2015, (2) feedback and trigger of household decision-making to ecological protection policies, and (3) management framework of a newly established national park.

## Materials and Methods

### Study Area

**FIGURE 1:**
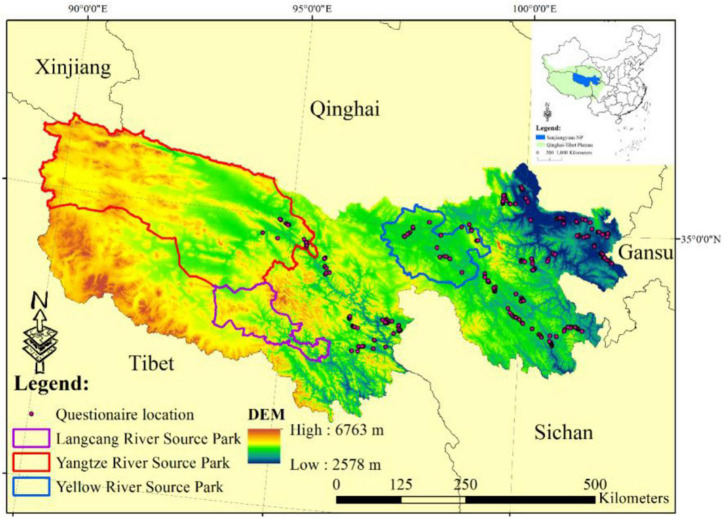
Location of the study area.

The SNP locates in the hinterland of the QTP (Figure 1). Due to the birthplace of the Yangtze River, the Yellow River, and the Lancang River, it is a critical area for water resource conservation, and its ecosystem is very sensitive and fragile (Zhai et al., 2020). It covers a 3.6 × 10^5^ km^2^ area, with an average altitude of 4,000 m (the altitude range is 2,582–6,815 m a.s.l.) (Ma et al., 2020). As the main parts of the SNP, Yangtze River Source Park, Yellow River Source Park, and Lancang River Source Park have their own position and orientation. The Yangtze River Source Park is positioned to protect alpine marshes, alpine meadows, and desert ecosystems; the Yellow River Source Park is positioned to protect the alpine wetland ecosystem; and the Lancang River Source Park is positioned to protect alpine valley landforms and the most abundant ecosystem types, including alpine meadow, alpine grassland, alpine shrub, and natural arbor forest (Xi et al., 2020). As the main ecosystem type, alpine grassland includes alpine meadow and alpine steppe, which account for approximately 76 and 23% of the total grassland area, which provides multiple ecological services for the upstream and downstream areas, such as food production, water conservation, and habitat support (Yu et al., 2021). Its annual precipitation is 262–773 mm and occurs mostly from June to September each year (Fang, 2013). The grass growth period is less than 3 months when there is no absolute frost-free period in the study area.

### Data Sources

#### Spatial Data Collection

Land use and cover change (LUCC) data in 2000, 2005, 2010, and 2015, distribution data of settlements (locations of cities, counties, and villages) and roads (national highways, provincial highways, county roads, and village roads), and river data (vector) were provided by the “National Tibetan Plateau Data Center.”^1^ All LUCC data were raster with a resolution of 30 m × 30 m. The Digital Elevation Model (DEM) data with a 30-m spatial resolution were downloaded from the United States Geological Survey (USGS).^2^ Historical weather data from the Climatic Research Unit (CRU) database (CRU, 2008) at 1° and 0.5° resolution for the years 2000–2015 were currently included with model files available for download from the G-Range model website (NREL, 2013; Sircely et al., 2019). All spatial data need to be transferred to ASCII data with a 0.25° × 0.25° resolution.

#### Questionnaire Investigation

We conducted a field survey to obtain 503 questionnaires with the pastoral interview of households from 2013 to 2015 in the SNP. According to the grazing intensity, alpine grassland type, and administration, 13 counties of four Tibetan autonomous prefectures were selected as the main interview areas, such as Yushu County, Zaduo County, and Qumalai County of Yushu Tibetan Autonomous Prefecture; Maqin County, Maduo County, Dari County, Banma County, Jiuzhi County, and Gande County of Guoluo Tibetan Autonomous Prefecture; Xinghai County and Tongde County of Huangnan Tibetan Autonomous Prefecture; and Zeku County and Henan Mongolian Autonomous County of Hainan Tibetan Autonomous Prefecture. The main contents of the questionnaire were divided into the following five parts: family information (including member, education, and location), livestock (including the total number of yaks, Tibetan sheep, and horses), household incomes (i.e., salary, livestock trade, *Cordyceps sinensis* trade, etc.), expenditure (i.e., food, clothes, medical costs, etc.), and others (mainly including awareness of eco-compensation and eco-migration) (Table 1).

**TABLE 1:** Household variables represented in the DECUMA model.

Attribute	Class	Unit	Notes
Family	Member	Person	Classify by age and gender
	Education		Uneducated, primary education, middle school, high school
	Location	Degree	Longitude and latitude
Livestock	Tibetan sheep	/	By sex
	Yak		
	Horse		
Income	Livestock trade	RMB (Yuan/a)	/
	Livestock products		/
	*Cordyceps sinensis* trade		/
	Other incomes		Salary
Expenditure	Food		/
	Clothes		
	Medical cost for people		
	Medical cost for livestock		
	Transportation cost		Transportation costs between summer and winter pastures
	Education cost		/
	Cultural cost		Praying or blessing costs in temples
	Other costs		/
Others	Perception of relevant protection policies		
	Whether snow disasters cause livestock death		
	Triggers to trade livestock		

### Data Analysis

#### G-Range Model

The G-Range model coupled biogeochemical submodels from the soil organic matter model (CENTURY model) with submodels of dynamic populations for herbs, shrubs, and trees to support spatial simulation and forecasting in the rangeland ecosystems (Sircely et al., 2019). The G-Range model could capture interannual and intra-annual variation and directional shifts in essential ecosystem processes, as well as differences in ecosystem process rates and competition among major rangeland plant growth forms (Sircely et al., 2019). The G-Range model was performed on a monthly time step and was a spatially explicit model at grid-cell scale, with model inputs from spatial layers driving ecosystem processes and vegetation dynamics according to the location and rangeland type (biome) of the grid/cell (Sircely et al., 2019). Based on the total area of the study area, we selected 0.25° as the basic resolution to run the G-Range model which currently supported grid/cell resolutions of 1°, 0.5°, 0.25°, 0.167°, 0.1°, and 0.083°.

#### DEcisions Under Conditions of Uncertainty by Modeled Agents Model

**FIGURE 2:**
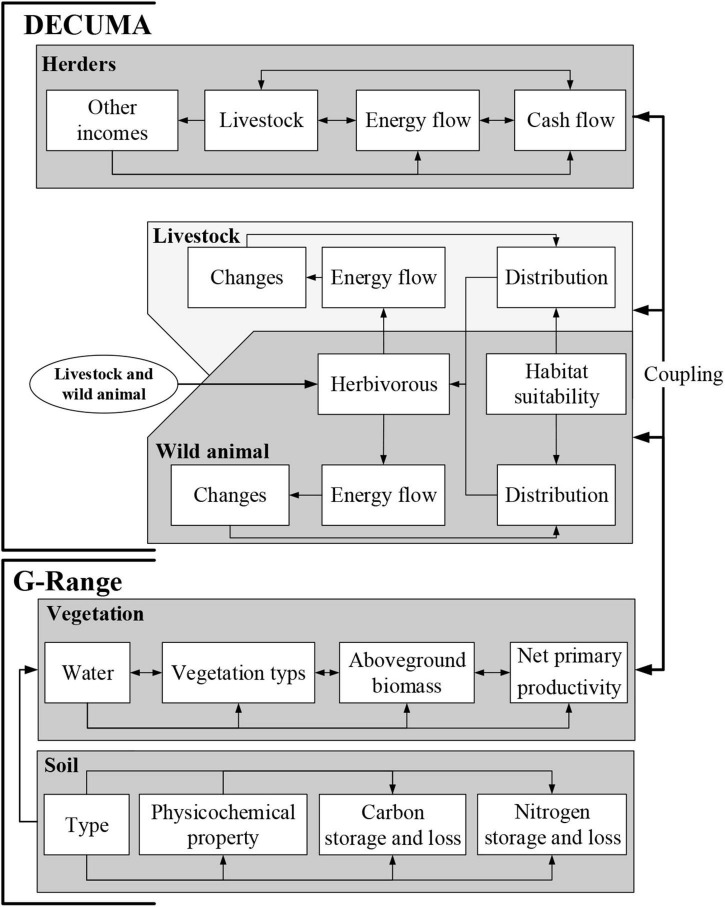
Detailed information flows linking G-Range model and DECUMA model.

As a spatially explicit household model, the DECUMA model was used to simulate decision-making and behaviors by pastoral households as they relate to ecosystem services (Boone et al., 2006). This model described the decision-making of local households to maintain their own survival and development in the process of uncertain future environmental changes. Measures reflecting livelihood of local households, such as livestock dynamics and holdings, energy flows where caloric gains from foods eaten were tallied and compared to energy needs, and cash flows, which included regularly scheduled income and expenses, as well as short term sales or purchases, were tracked (Figure 2; Boone et al., 2011). The DECUMA model linked to the G-Range model that could quantify ecosystem changes (e.g., forage availability) and could simulate the effects of livestock grazing on ecosystem (Thornton et al., 2003; Boone et al., 2011). With no fixed requirement for spatial scale, we simulated the effects of livestock grazing on ecosystem and feedbacks of ecosystem to livestock grazing coupling with G-Range model. The simulation process of DECUMA model mainly focused on the following two aspects: livestock distribution and dynamics and decision-making changes of local households. Among them, distribution and dynamics of livestock mainly included the following aspects: (1) simulated distribution of livestock (i.e., yak, Tibetan sheep, and horse) was determined based on the amount of edible forage provided by G-Range model and (2) total energy requirement was determined based on the available forage that livestock obtained, i.e., energy consumption (body weight changes of livestock). Population changes of livestock were closely related to birth and death rates of livestock, mainly by comparing the current number of livestock with the expected number of livestock in the future. Changes in the decision-making of local household were mainly based on the following aspects: (1) energy flow was the relative ratio of calories obtained from food and potential energy demands in each household; (2) cash flow mainly included economic income and expenditure of households; (3) livestock trade; and (4) cash needs were estimated for the next 3 months, which was related to the decision of the pastoralist to trade livestock (purchase, sale, or transfer of livestock) (Thornton et al., 2003; Boone et al., 2011).

## Results

### Changes in Habitat Suitability

**FIGURE 3:**
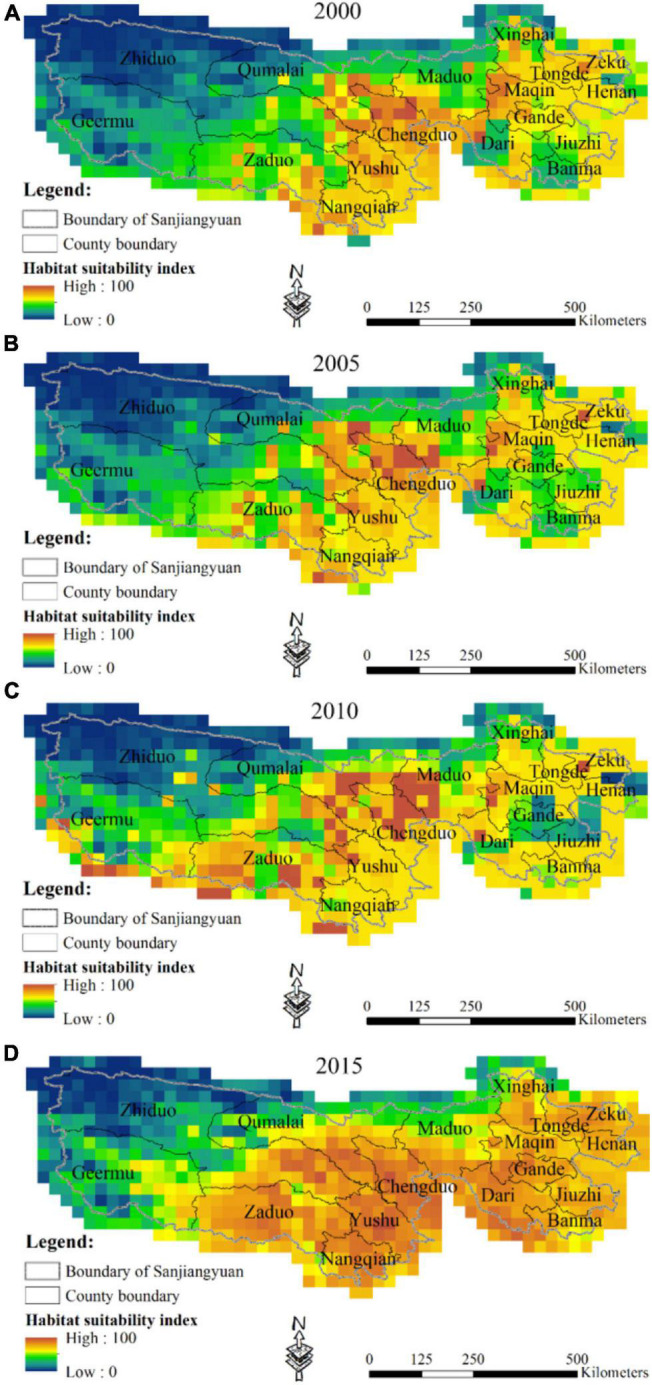
Habitat suitability index (HSI) changes in 1990 **(A)**, 2005 **(B)**, 2010 **(C)**, and 2015 **(D)**.

During the study period, unsuitable areas were mainly located at the western SNP where Yangtze River Source Park was located (Figure 3). Areas with high habitat suitability index (HSI) were mainly located in Chenduo County, Yushu County, and Maqin County in the middle and eastern SNP from 2000 to 2010 (Figures 3A–C). Areas with low HSI value appeared in the southeast SNP from 2000 to 2010 which transferred to areas with high and moderate HSI values in 2015. All unsuitable areas were located at Zhiduo County and Qumalai County in the northwestern SNP in 2015 (Figure 3D). With an area of approximately 2.4 × 10^5^ km^2^, about two-third of the SNP with high and moderate HSI value was in the central and eastern SNP in 2015. Compared with 2000, the HSI value of the eastern SNP showed an overall upward trend in 2015, while the change was not obvious in the central and western SNP. In 2000, unsuitable areas reached its maximum in the SNP. During the study period, areas with high and moderate HSI values gradually expanded westward in the SNP.

### Livestock Density Changes

**FIGURE 4:**
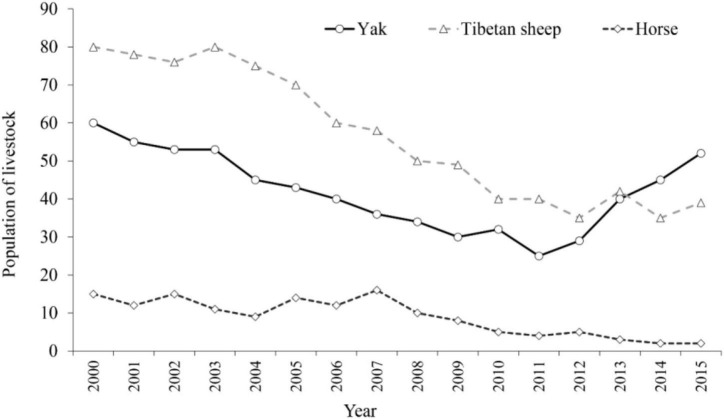
Annual changes of the average number of livestock (i.e., yaks, Tibetan sheep, and horses) from 2000 to 2015.

On the whole, the average number of Tibetan sheep, yaks, and horses in a household decreased from 80, 60, and 15 in 2000 to 39, 52, and 2 in 2015 in the SNP (Figure 4). Especially in 2011, the average number of yaks and Tibetan sheep had fallen by half to 25 and 40. The average number of Tibetan sheep and horses showed a significant decrease trend from 2000 to 2011, except for the average number of yaks, which recovered from 25 in 2011 to 52 in 2015. The average number of yaks held by local households was more than the average number of Tibetan sheep in 2015.

**FIGURE 5:**
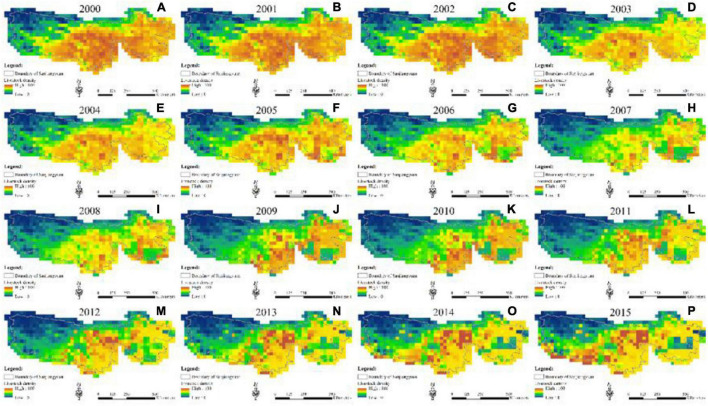
Livestock density changes in 2000 **(A)**, 2001 **(B)**, 2002 **(C)**, 2003 **(D)**, 2004 **(E)**, 2005 **(F)**, 2006 **(G)**, 2007 **(H)**, 2008 **(I)**, 2009 **(J)**, 2010 **(K)**, 2011 **(L)**, 2012 **(M)**, 2013 **(N)**, 2014 **(O)**, and 2015 **(P)**.

Spatial distribution of livestock density was basically consistent with distribution of habitat suitability of households in the SNP (Figure 5). The central and eastern SNP were the main distribution areas of livestock from 2000 to 2015. Since 2000, livestock density in the SNP showed a significant decrease trend. Areas with high livestock density gradually decreased during the study period. Especially in 2010, livestock density decreased most obviously in the study area. From 2010 to 2015, livestock density still maintained decrease trend in the whole nation park. However, the livestock density showed increase trend in the central part of the SNP (e.g., Maduo County, Chenduo County, Yushu County, and Nangqian County). Particularly, in 2015, the livestock density in Maduo County (Yellow River Source Park) showed a sharp increase trend (Figure 5P). Since 2007, the livestock density decreased significantly, mainly in Maqin County, Gande County, and Jiuzhi County of Guoluo Tibetan Autonomous Prefecture which were mainly in the eastern SNP (Figures 5H–P).

### Feedbacks to Ecological Protection Policies

**FIGURE 6:**
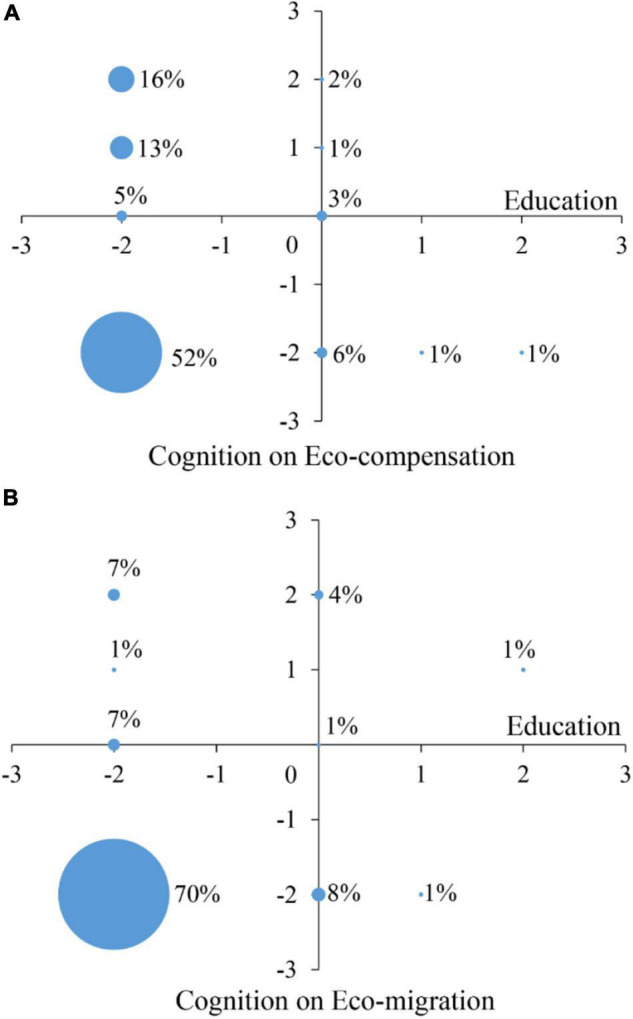
Cognition on eco-compensation **(A)** and eco-migration **(B)** of households with different education levels in the SNP. Education level: -2 stands for uneducated; -1 stands for temple education; 0 (zero) stands for primary school; 1 stands for middle school; and 2 stands for high school or above. The cognition on eco-compensation and eco-migration policies: -2 stands for against; -1 stands for mixed; 0 (zero) stands for no idea; 1 stands for medium; and 2 stands for support.

Local households with different education levels had different cognitions to ecological protection policies (Figure 6). A total of 52% uneducated households opposed eco-compensation policy to reduce the number of livestock, and 16% of uneducated households supported to adopt eco-compensation policy and accepted livestock reduction to protect alpine grassland (Figure 6A). There were 6% of local households with primary education level who opposed the implementation of eco-compensation policy, while only 2% of local households supported. However, households with a high school education level and above (including college education or university education) also opposed the eco-compensation policy.

Notably, 70% of uneducated households strongly opposed the implementation of eco-migration policy and thus to change their tradition and nomadic lifestyle, while only 7% of the uneducated households supported the eco-migration policy in the SNP (Figure 6B). Of note, 8% of households with primary school education level and 1% of households with middle school education level opposed this policy. Only 1% of households with high school education level and above (including college education or university education) did not oppose eco-migration policy.

More than a half (55.2%) of local households did not adopt any protection measures to protect their pastures and 37.8% of those adopted livestock reduction in 2015 (Table 2). Only 4% of local households selected grazing prohibition to protect alpine grassland.

**TABLE 2:** Obligation of local households on protecting alpine grassland linked to protection policies.

Grassland protection measures	Number of households	Percentage (%)
No actions	278	55.2
Livestock reduction	190	37.8
Grazing prohibition	20	4.0
Others	15	3.0
Total	200	100%

### Triggers of Household Decision-Making

**FIGURE 7:**
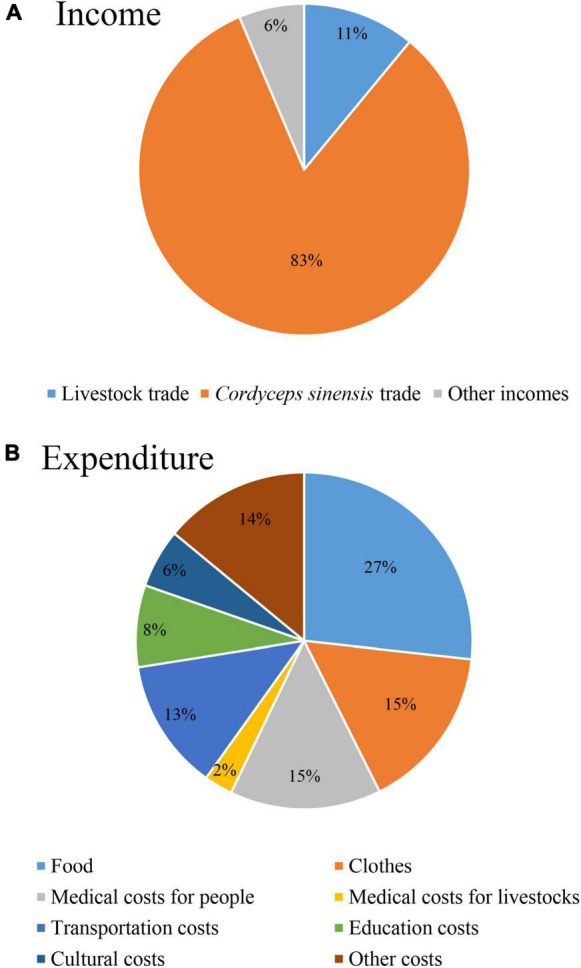
Income **(A)** and expenditure **(B)** of local households in the SNP.

The average annual income of local households was 11,374 yuan, and the average annual expenditure was 4,441 yuan, and thus the net income of local households was 6,933 yuan (≈1,000 dollars). Income of *Cordyceps sinensis* trade accounted for 83% (9,398 yuan) of the total income of household, income of livestock trade accounted for 11% (1,249 yuan), and other income accounted for 6% (727 yuan) in 2015 (Figure 7A). Food cost accounted for the largest proportion of 27% (1,188 yuan), followed by clothes cost and medical cost, both accounted for 15% (Figure 7B). Transportation, education, and culture costs (including praying and blessing costs in temples) accounted for 13 (560 yuan), 8, and 6%, respectively. More than half (53.7%) of households never chose to trade livestock for maintaining their livelihood. When local households owed 65,000 yuan (≈10,000 dollars) in debts (critical value of livestock trade), they chose to sell livestock to support their livelihood.

## Discussion

### Effects of Ecological Protection Policies

Our results indicated that livestock density distribution was basically consistent with the distribution of habitat suitability of local households in the SNP. Habitat deterioration was not improved at the beginning of Sanjiangyuan National Nature Reserve establishment in 2000, but habitat suitability was greatly improved after the implementation of alpine grassland protection policies in 2015. Most of the Yangtze River Source Park was an unsuitable area for local households, which was mainly located at Kekexili National Nature Reserve and listed as a representative of no man’s land in China. Most areas with high and moderate HSI values were located at Yellow River Source Park and Lancang River Source Park, which gradually expanded westward in the SNP from 2000 to 2015. Despite the strict implementation of eco-migration policy in the Yellow River Source Park, livestock density was still more concentrated and not in decline, especially in Maduo County. Although local households have moved out of this reserve and settled down, they returned back to their pasture for livestock grazing in the summer. This indicates that the implementation of eco-migration policy has not achieved the effect of alleviating the grazing pressure and protecting the alpine grassland ecosystem.

The average number of horses decreased sharply during the study period, which was not the main livestock held by local households, with the development of transportation infrastructure in the SNP. The average number of yaks held by local households was more than the number of Tibetan sheep in 2015. With the implementation of alpine grassland protection policies, local households will selectively reduce livestock, especially decreasing the number of Tibetan sheep, while the number of yaks (especially the number of female yaks) remains stable, because yaks can provide more dairy products (shortening, yogurt, etc.) and other husbandry products to maintain a livelihood. It indicates that implementation of eco-compensation only changes livestock structure and has no good effects on reducing the livestock population in the SNP.

### Feedback and Trigger

Cognition of local households toward alpine grassland protection policies (i.e., eco-compensation and eco-migration) changed during the study period. Local households differ in their education levels and do have significant feedbacks to ecological protection policies, practical schedules in livestock grazing, and managing their pastures (Yang et al., 2020). The proportion of local households who supported the eco-compensation policy increased from 54% (42% fully supported the policy and 12% moderately supported) in 2013 to 70% (50% fully supported the policy and 20% moderately supported) in 2015. The percentage of local households who opposed the implementation of eco-compensation policy decreased from 41% in 2013 to 26% in 2015. It indicates that local households in the national park gradually begin to accept eco-compensation policy. In 2013, only 18% of local households supported the eco-migration policy (16% fully supported it and 2% moderately supported it), and 76% opposed it. In 2015, the number of households who supported the eco-migration policy increased to 34% (20% of households fully supported the policy and 14% of herdsmen moderately supported it), while the number of households who opposed eco-migration policy decreased to 60%. Therefore, local households gradually begin to accept the eco-migration policy. Although more and more households are beginning to support eco-migration policy, most of the households still have against the implementation of eco-migration during the study period. With synchronous implementation of eco-compensation and eco-migration, cognition and acceptance of local households of eco-compensation policy are significantly higher than that of eco-migration policy.

Income of local households primarily derives from *Cordyceps sinensis* collection, traditional livestock husbandry, policy allowance, and sporadic side jobs in the SNP (Guo et al., 2021). Incidents of wildlife injuring people and livestock occur, which poses a risk to the life, property, and safety of local households, without any reasonable compensation standards or safeguard measures in place (Guo et al., 2021). Local households attribute alpine grassland conditions and observe their own management practices to their pastures, rotation grazing in winter and summer pastures, livestock reduction, and grazing prohibition (Yeh et al., 2017). Medical costs of livestock accounted for only 2% of total expenditure. Due to the implementation of eco-compensation, local governments provide more free veterinary medicines and veterinary services to local households who can reduce living costs. Livestock grazing is the basic condition for local households to survive and develop. To trade livestock and reduce its population are not only related to the survival and development of local households but also affect the formulation of alpine grassland protection policies. Therefore, analyzing the critical points of livestock trade is particularly important to balance human wellbeing and ecosystem protection. In this research, owing 65,000 yuan (≈10,000 dollars) in debts is the critical point (trigger) to trade livestock for supporting their livelihood by local households. In practice, this trigger should be considered in designing and planning eco-compensation policy by national park managers and local government policymakers.

### Effective Management Framework

The QTP is an integral ecological functional area in China and plays an important role in national ecological security shelter of China (Fan and Fang, 2020). The establishment of national parks is an important component of the ecological identity of China and an essential measure to achieving ecological civilization through natural-ecological protection and socioeconomic development (Guo et al., 2021). The provincial government of Qinghai established Sanjiangyuan provincial nature reserve in 2000 and officially approved it as a national nature reserve in 2003. Accompanied by the ecological civilization system reform, the SNP was one of the national park pilots in China in 2015 and was established as the first national park of China in 2021 (Xi et al., 2020; Guo et al., 2021). It is also of particularly important to end a national park pilot program and to establish an official national park, which has aimed to protect the local ecosystem for improving ecosystem services of alpine grassland, restoring biodiversity, and achieving breakthroughs in the management system on the QTP (Guo et al., 2021; Ma et al., 2021; Su et al., 2021). More importantly, effective management and law enforcement system of a newly established national park are key urgent problems that demand a prompt solution (Su et al., 2021). Based on more technical supports from scientific research, adaptive management on the carrying capacity of alpine grassland and its utilization strategy should be strengthened by managers and policymakers who wish to achieve policy outcomes that benefit both local households and alpine grassland may do well to fully consider household decision-making with existing knowledge, education levels, cultural context, and management practices of local herdsmen (Yang et al., 2020). Specifically, managers and policymakers need to understand that limiting livestock grazing to a sustainable level is a more reasonable approach if overgrazing exists based on carrying capacity of alpine grassland (Zhao et al., 2020).

**FIGURE 8:**
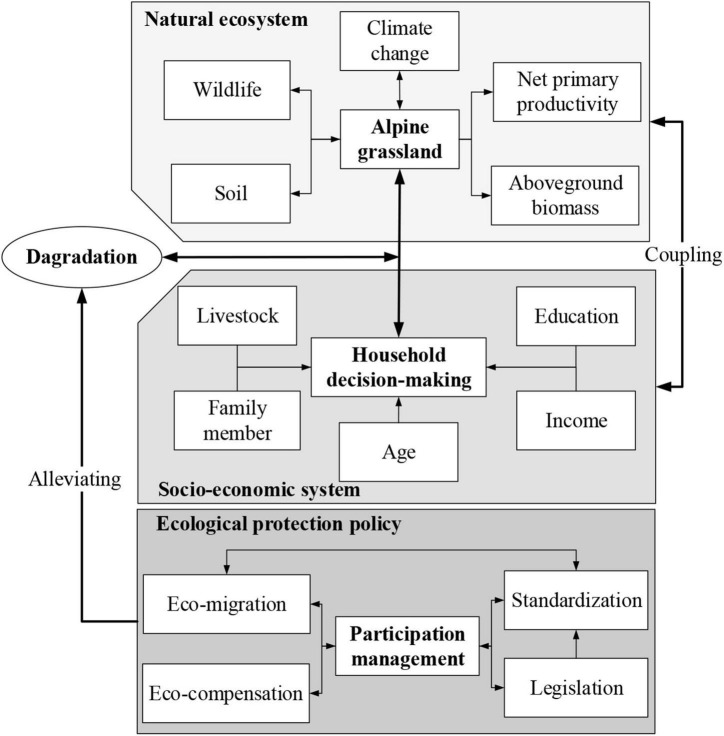
Management framework to alleviate alpine grassland degradation and to enhance the wellbeing of local households based on coupling natural and socioeconomic systems.

Differences in stakeholder values are at the heart of protection policies, with dissimilarities in objectives fueling the challenges of managing complex, dynamic systems (Mason et al., 2018; St John et al., 2021). In this research, we propose a new management framework of the SNP coupling natural ecosystem with socioeconomic system, which needs to fully consider the decision-making of households and their feedback and trigger to ecological protection policies (Figure 8). Managers and policymakers should carry out household-based participation management which needs to be adjusted in real time according to different feedback and trigger of household decision-making to face future challenges. Conventionally, this management framework needs to focus on reducing pressures that affect alpine grassland, such as conflicts between biodiversity conservation and natural resources utilization. Meanwhile, it should also have the ability to balance short-term needs with long-term requirements for the future sustainability of alpine grassland and to integrate the dynamic and complex relationships between local herdsmen and alpine grassland (Thiault et al., 2018). In total, it should be a more complex and integrative approach coupling humans with nature rather than a normal method in biodiversity conservation and natural resource management.

## Conclusion

This research indicates that livestock density distribution is basically consistent with the distribution of habitat suitability of households in the SNP. The implementation of alpine grassland protection policies can decline habitat deterioration and greatly enhance habitat suitability of local households. Local households have different cognitions to eco-compensation and eco-migration policies due to their different education levels. Moreover, there is a trigger that local households make decisions to trade livestock to support their livelihood. Our results suggest that it is necessary to design a new framework to effectively manage the newly established national park. This framework should fully consider feedback and trigger of household decision-making which can guide policymakers to plan ecological protection policies for maintaining sustainable development of alpine grassland.

## Data Availability Statement

The original contributions presented in the study are included in the article/supplementary material, further inquiries can be directed to the corresponding author/s.

## Author Contributions

XS, SD, and GL designed the research. YL, YS, and HC performed the analysis. XS drafted this article. All authors contributed to the interpretation of this article, approved the submitted version, and collected data in the field.

## Conflict of Interest

The authors declare that the research was conducted in the absence of any commercial or financial relationships that could be construed as a potential conflict of interest.

## Publisher’s Note

All claims expressed in this article are solely those of the authors and do not necessarily represent those of their affiliated organizations, or those of the publisher, the editors and the reviewers. Any product that may be evaluated in this article, or claim that may be made by its manufacturer, is not guaranteed or endorsed by the publisher.

## Footnotes

1
http://data.tpdc.ac.cn

2
http://earthexplorer.usgs.gov/
